# Ultrasensitive and rapid count of *Escherichia coli* using magnetic nanoparticle probe under dark-field microscope

**DOI:** 10.1186/s12866-018-1241-5

**Published:** 2018-09-03

**Authors:** Haixu Xu, Fang Tang, Jianjun Dai, Chengming Wang, Xin Zhou

**Affiliations:** 1 Institute of Comparative Medicine, Jiangsu Co-innovation Center for Prevention and Control of Important Animal Infectious Diseases and Zoonoses, Joint International Research Laboratory of Agriculture and Agri-Product Safety, the Ministry of Education of China, Yangzhou University, Yangzhou, 225009 China; 2 Key Laboratory of Animal Bacteriology, Ministry of Agriculture, College of veterinary medicine, Nanjing Agricultural University, Nanjing, 210095 China; 3 Department of Pathobiology, College of Veterinary Medicine, Auburn University, 268 Greene Hall, 1130 Wire Rd, Auburn, AL 36849-5519 USA

**Keywords:** Rapid enumeration, *Escherichia coli*, Magnetic nanoparticle, Dark-field microscope

## Abstract

**Background:**

*Escherichia coli (E. coli)* is one of the best-known zoonotic bacterial species, which pathogenic strain can cause infections in humans and animals. However, existing technologies or methods are deficient for quickly on-site identifying infection of *E. coli* before they breakout. Herein, we present an ultrasensitive and on-site method for counting *E. coli* using magnetic nanoparticle (MNP) probe under a dark-field in 30 min.

**Results:**

The antibodies functionalized MNP, binding to *E. coli* to form a golden ring-like structure under a dark-field microscope, allowing for counting *E. coli*. This method via counting MNP-conjugated *E. coli* under dark-field microscope demonstrated the sensitivity of 6 CFU/μL for *E. coli* detection. Importantly, due to the advantages such as time-saving (only 30 min) and almost free of instrument (only require a portable microscope), our MNP-labeled dark-field counting strategy has the potential of being a universal tool for on-site quantifying a variety of pathogens with size ranges from a few hundreds of nanometers to a few micrometers.

**Conclusion:**

In summary, the MNP-labeled dark-field counting strategy is a rapid, simple, sensitive as well as low-cost assay strategy, which has the potential of being a universal tool for on-site quantification of micrometer-size pathogens like *E. coli*.

**Electronic supplementary material:**

The online version of this article (10.1186/s12866-018-1241-5) contains supplementary material, which is available to authorized users.

## Background

*E. coli* is a rod-like bacterium and the genus *Escherichia* contains mostly motile Gram-negative bacilli in the family of *Enterobacteriaceae*. Most of the *E. coli* strains are not harmful to humans but some strains possess pathogenic virulence, which increase the ability to adapt new niches and allow them to cause broad spectrum of diseases [1]. In recent years, the foodborne diseases emerged as major concern in public [2]. Therefore, rapid, inexpensive and sensitive methods are required to detect the *E. coli* infection.

During last decades, several molecular and immunological methods have been developed to detect *E. coli.* Such as polymerase chain reaction (PCR), real-time PCR, multiplexed PCR, loop-mediated isothermal amplification, Pulsed-Field Gel Electrophoresis, Gene-chip, next generation sequencing, enzyme linked immunosorbent assay (ELISA) and immune chromatography. Despite the fact of these approaches are powerful, they have drawbacks such as laboriousness, high-cost, complexity, and requirement of expensive instruments or reagents. Most importantly, they are not able for on-site assay [3–11]. Recently, the biosensor technology plays an important role in the detection of pathogenic bacteria because they have great potential to meet the practical need of rapid, sensitive, easy-to-use, and low-cost [12, 13]. Biosensor technology implemented with the use of nanoparticles (NPs) tags and also other nanomaterials offers benefits compared with traditional methods in terms of time, sensitivity and simplicity [14–17].

**Fig. 1:**
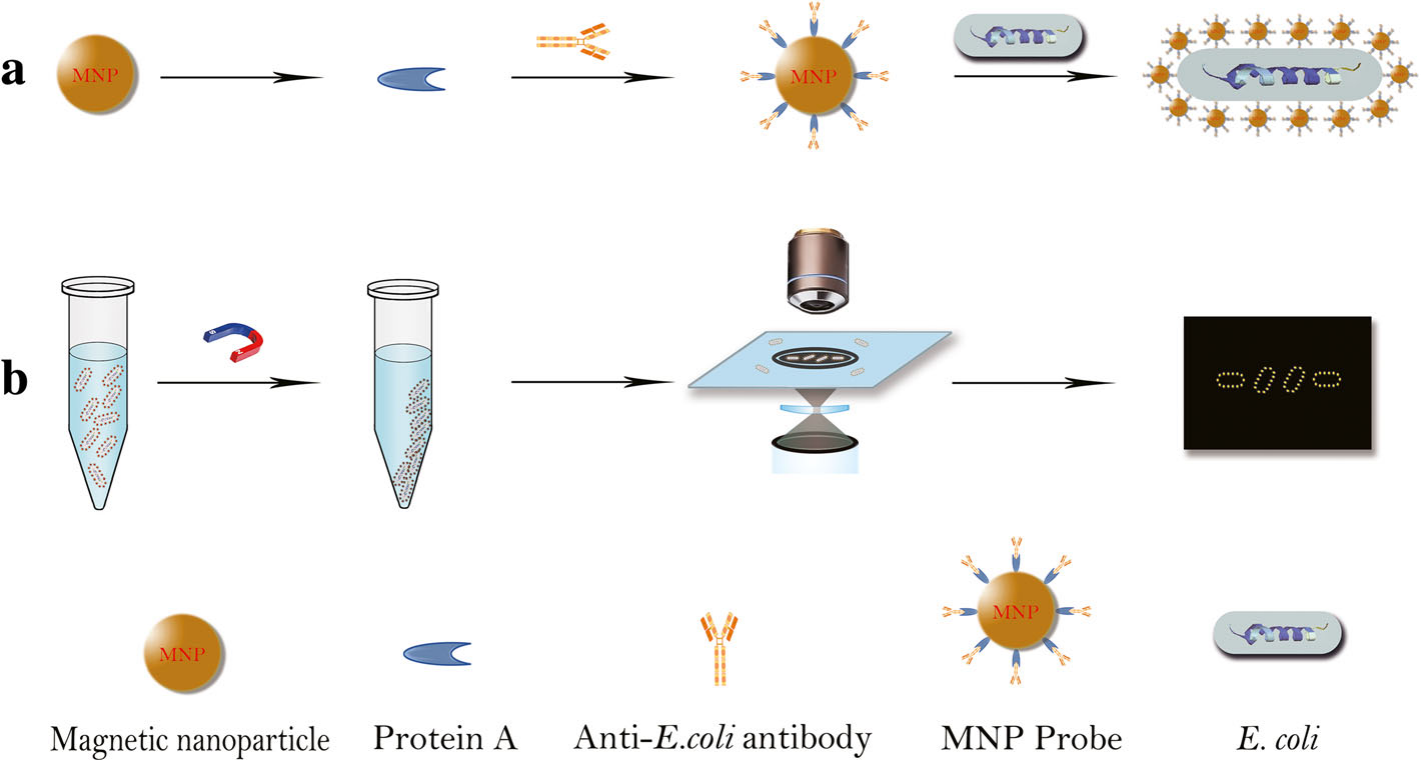
Schematic diagram of counting *E. coli* under a dark-field by hatting them golden ring structure with MNP probes. **a** The MNP probes were obtained by incubating anti-*E. coli* antibodies and the MNP under gently shaking for 4 h at room temperature, followed by washing three times with PBS. **b** *E. coli* samples were first mixed with MNP probes to develop probe-*E. coli* complexes, followed by magnetic separation for counting with dark-field microscope

In this study, we demonstrated a quick and sensitive counting of *E. coli* at low concentrations without bacteria pre-enrichment by employing a MNP probe combined with dark-field microscope. The schematic diagram of our MNP-labeled dark-field counting strategy is shown in Fig. 1.

## Results

### Validation of MNP probes

**Fig. 2:**
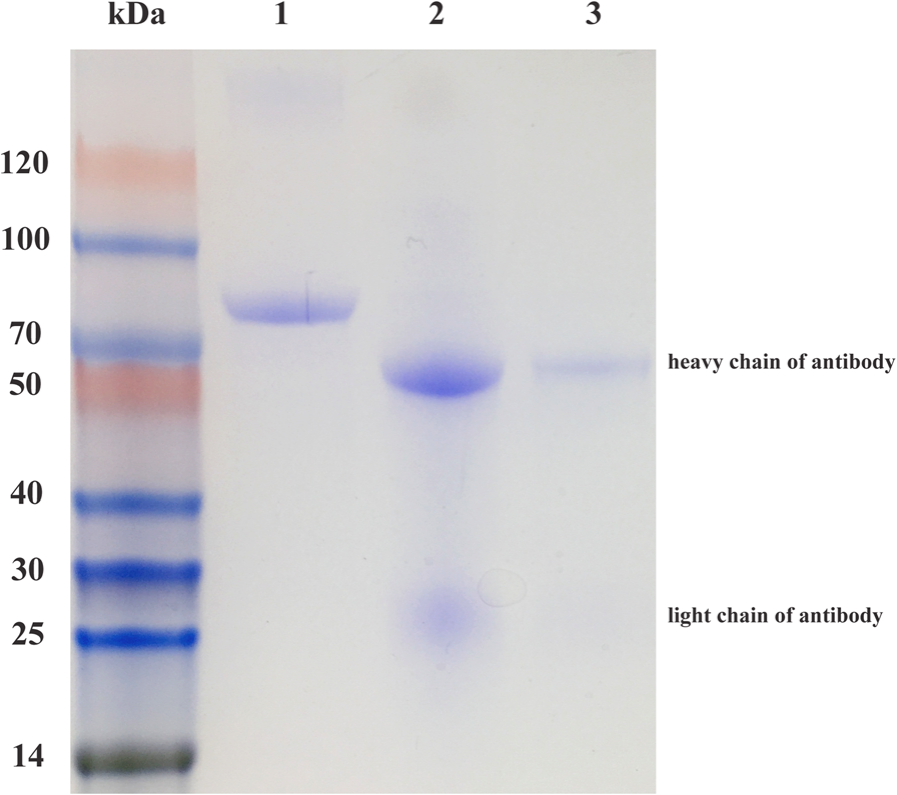
SDS-PAGE analysis of prepared MNP probes. Column 1: 10 μL of as-synthesized MNP; Column 2: 5 μg anti-*E. coli* antibody; Column 3: 10 μL of MNP probes

The electrophoresis result clearly showed the presence of two bands (a ~ 50 kDa band of antibody heavy chain and a ~ 20 kDa band of antibody light chain) in MNP probes sample (Fig. 2, lane 3), indicating successful bio-functionalization of MNP probes.

### Detection of *E. coli* cultured in laboratory with our counting strategy

**Fig. 3:**
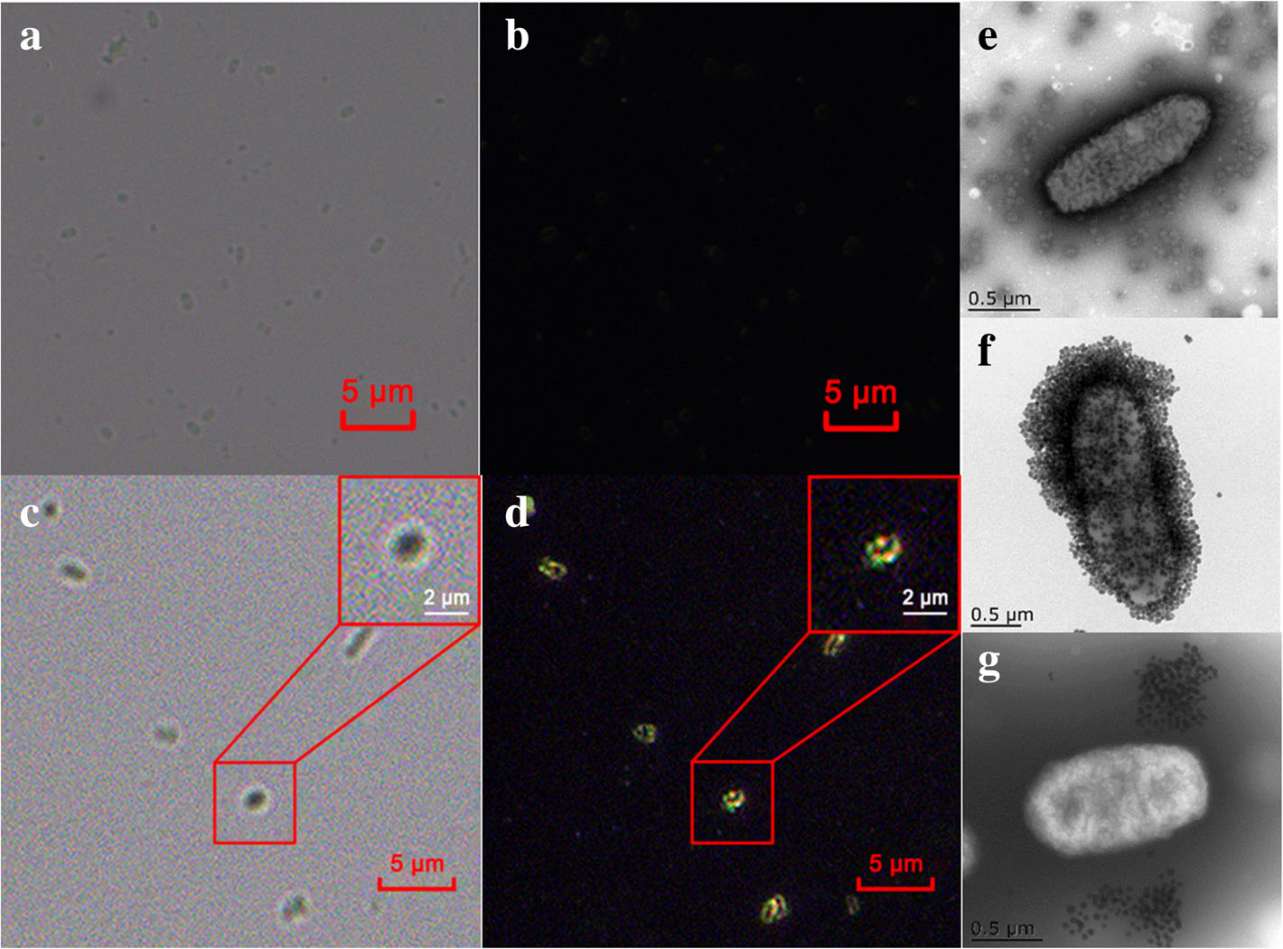
Microscope images of *E. coli* at different conditions. **a** *E. coli* without MNP probes under a bright field; **b** *E. coli* without MNP probes under a dark-field; **c** *E. coli* mixed with MNP probes under a bright field; **d** *E. coli* mixed with MNP probes under a dark-field; **e** Transmission Electronic Microscope (TEM) image of *E. coli* mixed with naked MNPs. **f** TEM image of *. coli* with MNP probes. **g** *Salmonella* mixed with MNP probes; Red box in the upper right corner of d and e is the enlargement of the selected area, respectively

The results show that each bacteria was encircled with MNP probes to form dim bacilliform structures under light field (Fig. 3c) and bright gold ring structure under dark-field microscope (Fig. 3d), which was also confirmed by TEM (Fig. 3f). On the contrary, *E. coli* mixed with MNP without anti-*E. coli* antibodies were observed under a bright and dark-field microscope. *E. coli* were dim and indistinct under bright field (Fig. 3a) and were completely invisible under dark field (Fig. 3b) due to no recognition between *E. coli* and MNP particles, which was verified by TEM (Fig. 3e). The Fig. 3g clearly showed that *Salmonella* bacteria did not bind to MNP probes, which suggests that our MNP probes has a specific affinity to target species.

### Determination of limit of detection of our counting assay

**Fig. 4:**
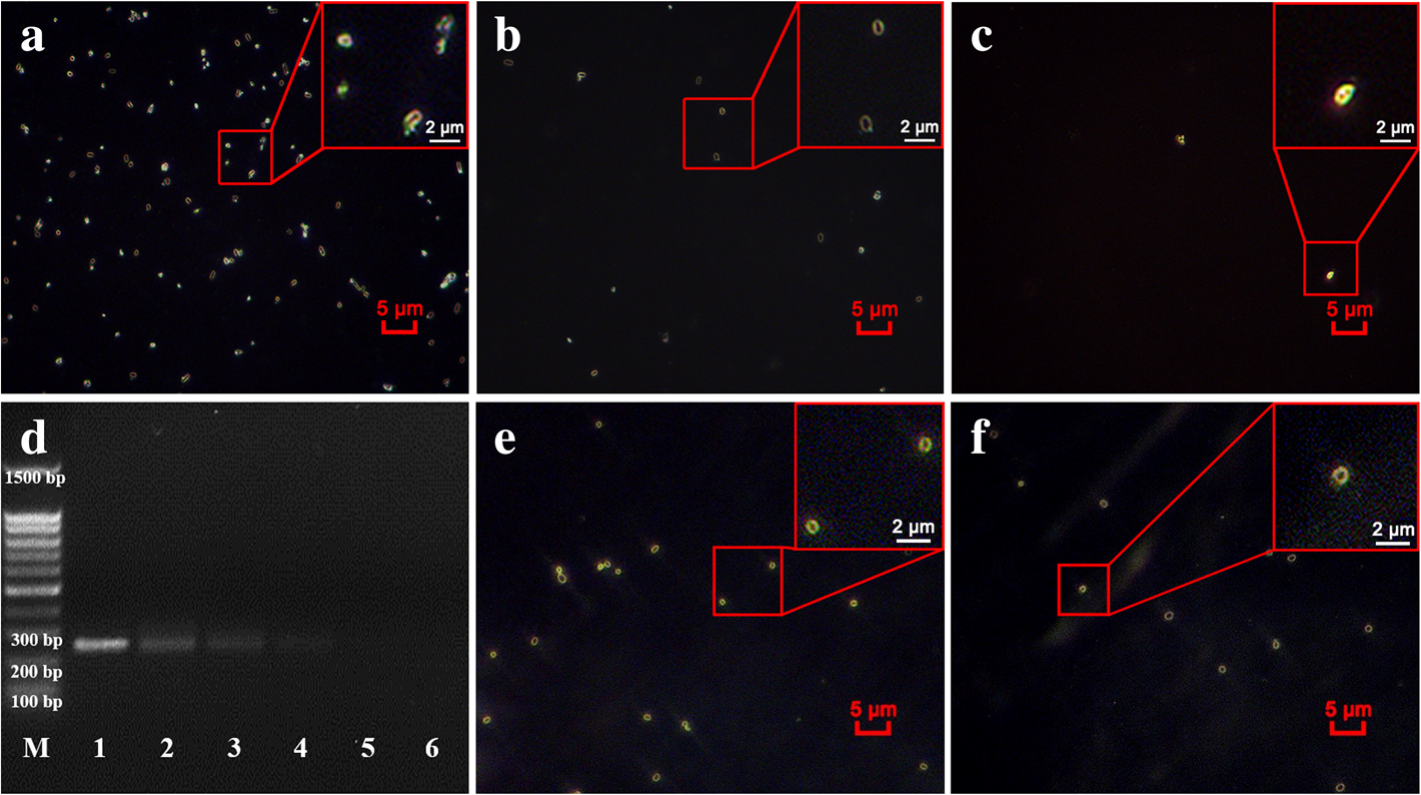
Sensitivity analysis of *E.coli* at different dilution concentrations and count of two real samples by our MNP-based strategy. **a** 10^3^ dilution of *E. coli* stock solution (equal to 10^2^ CFU/μL) captured by MNP probe; **b** 10^4^ dilution of *E. coli* stock solution (equal to 10 CFU/μL) captured by MNP probe; **c** 10^5^ dilution of *E. coli* stock solution (equal to 1 CFU/μL) captured by MNP probe. **d** PCR analysis of *E.coli* at different dilution concentrations. M: DNA Marker; Line 1: *E. coli* stock solution; Line 2–6: 10^1^, 10^2^, 10^3^, 10^4^ and 10^5^ dilution of *E. coli* stock solution, respectively; Red box in the upper right corner of **a**, **b** and **c** is the enlargement of the selected area, respectively. **e** A representative dark-field image of *E. coli* in ten-fold concentration dilute soup sample captured by MNP probes; **f** A representative dark-field image of *E. coli* in ten-fold concentration rice sample captured by MNP probes

Figures 4a (10^2^ CFU/μL) and 4b (10 CFU/μL) show that many monodisperse golden ring-like structures can be counted under dark-field. Even in Fig. 4c (1 CFU/μL of *E. coli* sample), we could also occasionally find one or two bright golden ring structure in a certain field of view. The limit of detection is defined as three times the signal-to-noise ratio, which is calculated by counting the number of golden ring-like *E. coli* found in 10 random fields of view of background experiment (naked MNP mixed with *E. coli*). Because 10 μL of sample solution covers the area of 200 field of views (each view area equal to 100 μm^2^) and the background experiments showed that one dim ring-like structure could be occasionally found in 10 random field of views, LOD of our counting method could be defined as 6 CFU/μL. The maximum quantity of *E. coli* in single field of view can be used to clearly count is approximated to 300 CFU, the suitable concentration range by our MNP-based counting method is from 6 to 6000 CFU/μL. The results show the LOD of PCR in this study is 100 CFU/μL (Fig. 4d, line 4) because the band from 10 CFU/μL sample is invisible to the naked eye. These results indicate that the theoretical sensitivity of our counting strategy is 15 times more sensitive than that of PCR technique. Moreover, it is worth to mention that, strictly speaking, no false positive happened using our MNP-based dark-field microscopy counting method because no bright gold ring structures formation in the absence of target pathogens.

### Detection of real samples with MNP-labeled dark-field counting strategy

Two food samples that were exposed to the air for 24 h in our lab were quantified with our MNP-labeled dark-field counting method. As shown in the Table 1, 163 ± 16 CFU/10 μL from soup sample and 126 ± 14 CFU/10 μL from rice sample could be obtained by counting the clones in petri dishes (The raw counting images were shown in Additional file 1: Figures. S1 and S2). Using our counting method, 142 ± 14 CFU/10 μL and 112 ± 8 CFU/10 μL could be counted, respectively (the raw dark-field images by our counting strategy were shown in Additional file 1: Table S1). The high coincidence rate (~ 87%) compared to the clone counting method and efficient detection time of 30 min suggest that our strategy will be a more advantageous method of enumeration of *E. coli* in real samples than the traditional method that normally takes 10 h for analysis. Figure 4e and Fig. 4f are the representative dark-field images of MNP-*E. coli* from the ten-fold concentration dilute soup and rice, respectively (Additional file 1: Figures. S3 to S8).

**Table 1:** Detection of real samples by traditional colony counting and MNP-labeled dark-field counting strategy

Samples	Colony counting method (CFU of 10 μL of sample)	Our counting strategy (CFU of 10 μL of sample)
Dilute soup	163 ± 16	142 ± 14
Rice	126 ± 14	112 ± 8

Statistical analysis: The experimental counting results are reported as means ± SE of triplicate independent experiments. Ten fields of view were counted for each sample. Data were analyzed using the SPSS (Statistical Package for the Social Sciences) software (Version 13.0; SPSS, Inc., Chicago, IL)

## Discussion

Since the quantity of *E. coli* is used as a potential indicator for the presence of other pathogens in many samples [18], the number of *E. coli* is essential for evaluating the extent of enteric disease by pathogenic bacteria such as enterohemorrhagic strains of *E. coli* [19, 20] that contaminates food or water [20, 21]. Conventional identification methods for bacteria involves several steps: selective culture, Gram staining, as well as biochemical and/or serological tests, which are still considered as gold standard for *E. coli* detection [22]. However, the conventional assay is labor-intensive and often time-consuming and strenuous to obtain a confirmed result. Theoretically, the sensitivity of our MNP-labeled dark-field counting strategy can reach 1 CFU in 10 μL of sample solution because the structure of non-specific adsorption formation is far from the bright gold ring. However, considering the convenience of practical assay, the LOD of our counting method can be determined approximate to 6 CFU/μL. And the suitable concentration range by our MNP-based counting method is from 6 to 6000 CFU/μL. The wide range of detection (6 to 6000 CFU/μL) can cover a variety of *E. coli* samples even without concentration or dilution; therefore, our MNP-labeled dark-field counting strategy is an efficient technique for counting the *E. coli* from different samples.

In this study, a rapid, reliable and effective assay has been demonstrated for the detection of *E. coli* in samples with different concentrations. The sensitivity of the MNP-based counting method was thoroughly investigated and compared to the conventional PCR assay. The data showed that LOD of our counting method is down to 6 CFU/μL, which greatly exceeds the PCR assay (the LOD of PCR assay performed in this study is 100 CFU/μL). Owing to the advantages such as time-saving (~ 30 min) and almost free of instrument (only the requirement of a portable microscope), MNP-labeled dark-field counting strategy has the potential of being a universal platform for on-site quantifying a variety of pathogens in all sizes ranging from hundreds of nanometers to micrometers. The MNP-based counting method potentially allows for on-site use in the monitoring of water quality, food safety and environmental parameters.

## Conclusion

In summary, a rapid, simple, sensitive as well as low-cost assay has been developed for on-site enumeration of micrometer-size pathogens like *E. coli*.

## Methods

### Bacterial culture

*E. coli* O78 used in this study was a gift from Dr. Fang Tang (College of veterinary medicine, Nanjing Agricultural University). The bacteria were cultured in Luria Bertani (LB) medium and kept shaking at 220 rpm at 37 °C over night. Then the cells were harvested and washed for several times with 10 mM PBS solution (pH = 7.4). Optical density at 600 nm (OD_600_) was measured and adjusted to around 0.60, corresponding to bacteria concentration of 10^8^ CFU/mL.

### Preparation of MNP probes

To demonstrate the proof-of-concept above, the MNP probes were prepared as follows: 1) Protein A functionalized MNPs (Creative Diagnostics, USA) at diameter of ~ 50 nm were incubated with anti-*E. coli* polyclonal antibody (5 μg/μL) (PA1–7213, Thermo Fisher, USA) for 4 h at room temperature. 2) The MNP probes were washed three times with PBS by magnetic stand to remove the unattached *E. coli* antibodies and then were stored at 4 °C until use. 3) To investigate the successful modification of antibody, MNP probes were assayed using the sodium dodecyl sulfate−polyacrylamide gel electrophoresis (SDS-PAGE).

### Detection of *E. coli* with MNP-labeled dark-field counting strategy

With the achievement of prepared MNP probes, we next examined the feasibility of counting *E. coli* under dark-field microscope. The counting assay solution containing 50 μL of MNP probes (~ 8 × 10^11^ particles), 10 μL of *E. coli* (~ 1.0 × 10^6^ CFU) and 140 μL of PBS (pH 7.4) was gently mixed for 25 min at room temperature, and then immediately separated by magnetic stand. The resulting solution was further washed three times with PBS and subjected to dark-field microscopy and transmission electron microscopy (TEM, Tecnai 12, Philips, Holland). A *Salmonella* strain (BNCC103307, purchased from ATCC) was employed as a control to investigate the specificity of MNP probes.

### Investigation of sensitivity of MNP-labeled dark-field counting strategy

In addition, the sensitivity of the proposed method was subsequently investigated. A serial of *E. coli* samples cultured in LB medium at different concentrations were employed to investigate the limit of detection (LOD) using our MNP-based dark-field microscopy counting method. Briefly, the *E. coli* stock solution was diluted with PBS buffer into five samples at different concentration from 10^0^ to 10^4^ CFU/μL. 10 μL of *E. coli* sample solution was mixed with 50 μL of MNP probes and 140 μL of PBS (pH = 7.4) for 25 min, and then separated by magnetic stand, finally subjected to dark-field microscopy. Meanwhile, PCR assay was employed as a compared method to evaluate the sensitivity of our counting strategy. To this end, a pair of primers (forward primer: 5′-TAGGTATTCCTGTTGCGGAGTATAT-3′; reverse primer: 5′-TACTATACATCAAACCCTCAGCATT-3′) were designed to amplify a fragment of specific *E. coli* gene (6-phosphoguuconate dehydrogenase). PCR were performed according to standard procedure and 2 μL of each of *E. coli* samples at different concentration was used as template in each PCR reaction.

### Detection of real samples with MNP-labeled dark-field counting strategy

Furthermore, two real samples, one of which is a dilute meat soup and the other is a rice exposed to air for two days, were employed to demonstrated the availability of our MNP-labeled dark-field counting strategy. Firstly, two samples were washed with PBS under gently mixing for 5 min and then the supernatants were collected by centrifugation at 10,000 g for 3 min. 10 time-fold condensed sample solutions were prepared at the same time. 50 μL of condensed samples were mixed with 100 μL of anti-*E. coli* antibody functionalized MNP probe under gently shaking for 25 min at room temperature and then washed three times with PBS, followed by subjecting to dark-field microscopy for enumeration. In the meantime, the conventional colony enumeration method was employed as a comparison to evaluate the feasibility of our MNP-labeled dark-field counting strategy.

## Additional file

Additional file 1: Counting clones of dilute soup and rice samples in petri dishes. Dark-field images of real samples using the MNP-based counting method. Counting clones of dilute soup samples in petri dishes. Counting clones of rice samples in petri dishes. 10 random views of dark-field images of MNP-*E. coli* from the ten-fold concentration dilute soup. Three repetitions were performed. 10 random views of dark-field images of MNP-*E. coli* from the ten-fold concentration rice. Three repetitions were performed. The counting numbers were marked on respective dark-field images. (DOCX 666 kb)

## Abbreviations

CFU: Colony forming units
*E. coli*: *Escherichia coli*
ELISA: Enzyme linked immunosorbent assay
h: hour
LB: Luria Bertani
LOD: Limit of detection
min: minute
MNP: Magnetic nanoparticle
NPs: Nanoparticles
PBS: Phosphate buffered solution
PCR: Polymerase chain reaction
rpm: revolution per minute
SDS-PAGE: Sodium dodecyl sulfate−polyacrylamide gel electrophoresis
TEM: Transmission electron microscopy
