# Supplementary material for: Ultrasensitive and rapid count of Escherichia coli using magnetic nanoparticle probe under dark-field microscope
Source: BMC Microbiol. 2018 Sep 3;18:100. doi: 10.1186/s12866-018-1241-5 (PMC6122661; doi:10.1186/s12866-018-1241-5)
Supplement: Supplementary file 1 — Counting clones of dilute soup and rice samples in petri dishes. Dark-field images of real samples using the MNP-based counting method. Counting clones of dilute soup samples in petri dishes. Counting clones of rice samples in petri dishes. 10 random views of dark-field images of MNP-E. coli from the ten-fold concentration dilute soup. Three repetitions were performed. 10 random views of dark-field images of MNP-E. coli from the ten-fold concentration rice. Three repetitions were performed. The counting numbers were marked on respective dark-field images. (DOCX 666 kb) [file 12866_2018_1241_MOESM1_ESM.docx]

**Figure S1. Counting clones of dilute soup samples in petri dishes.**

**
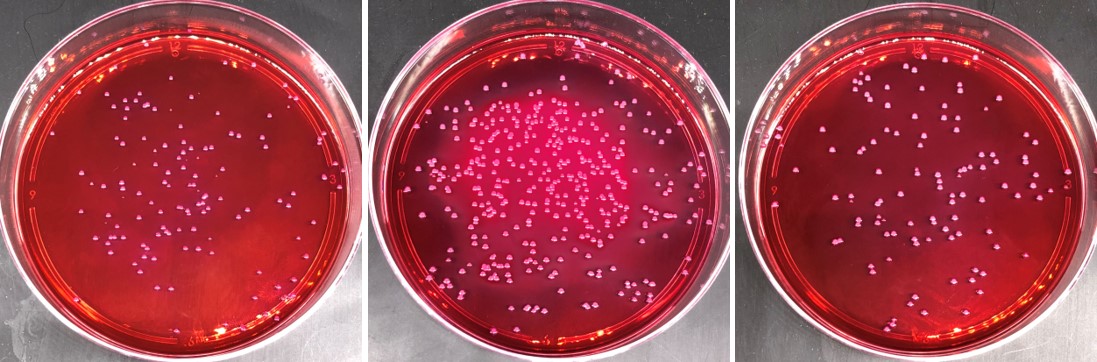
**

**Figure S2. Counting clones of rice samples in petri dishes.**

**
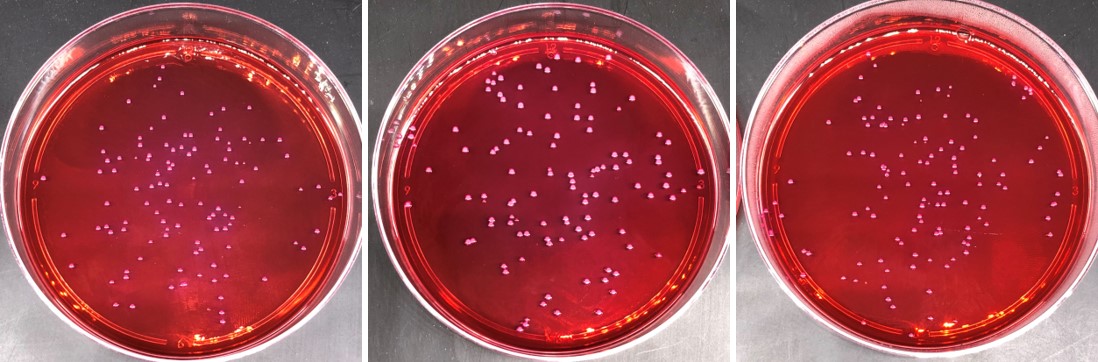
**

**10 random views of dark-field images of MNP-*E. coli* from the ten-fold concentration dilute soup. Three repetitions were performed.**

**Figure S3.**

**
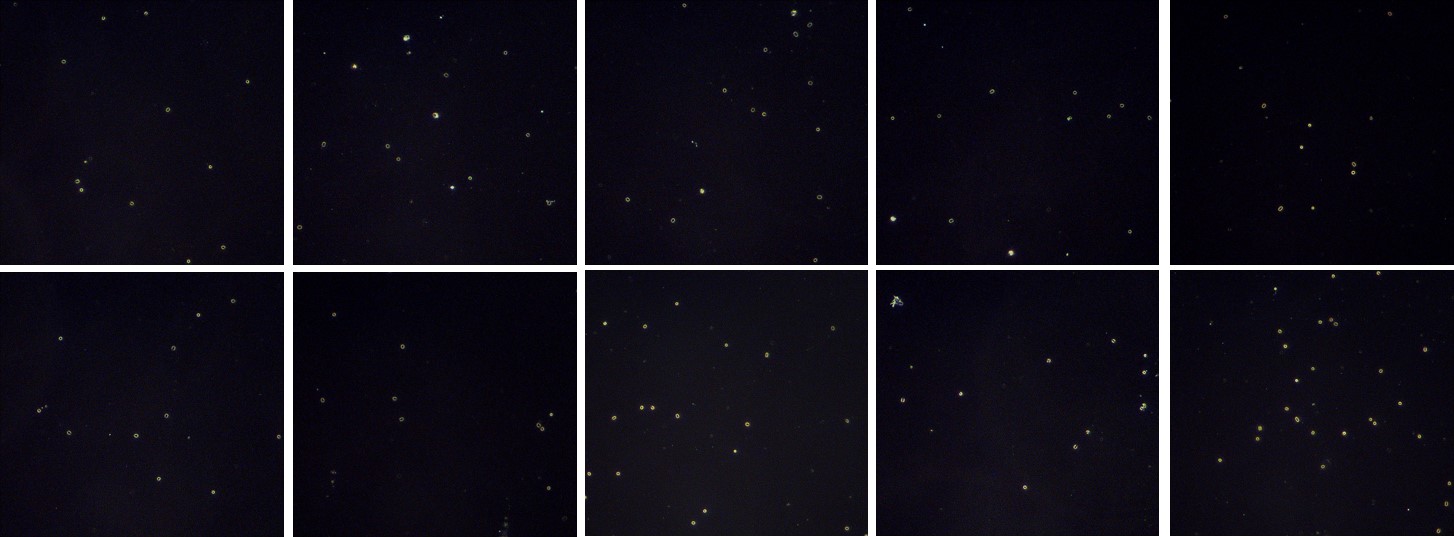
**

**Figure S4.**

**
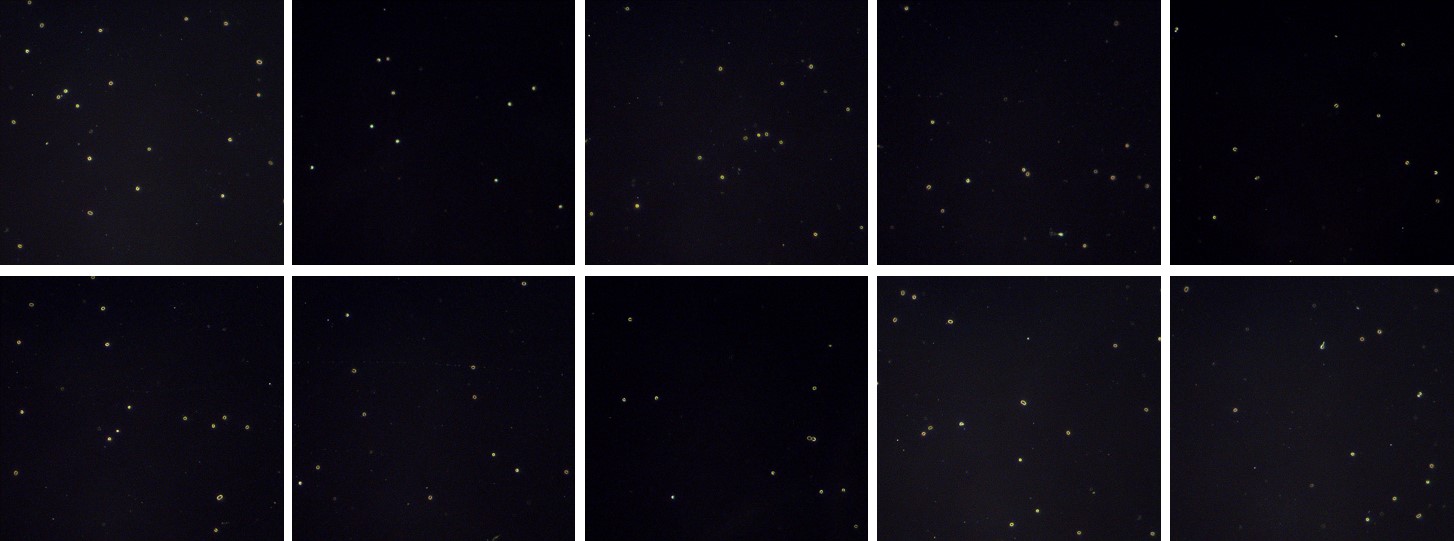
**

**Figure S5.**

**
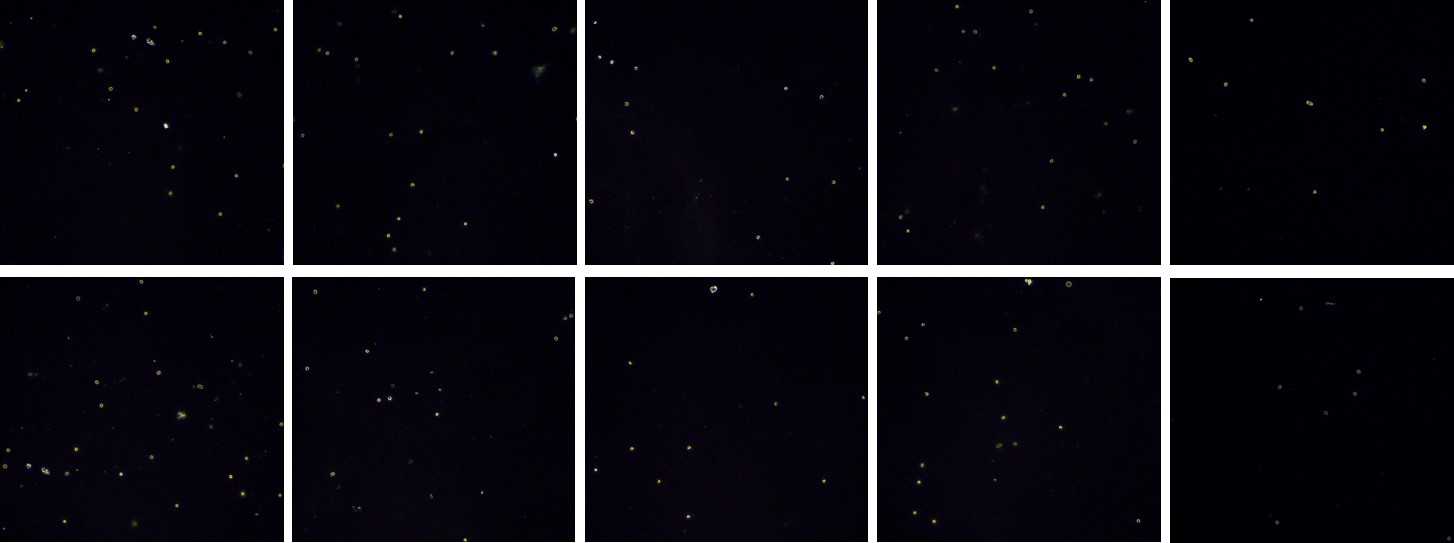
**

**10 random views of dark-field images of MNP-*E. coli* from the ten-fold concentration rice. Three repetitions were performed.**

**Figure S6.**

**
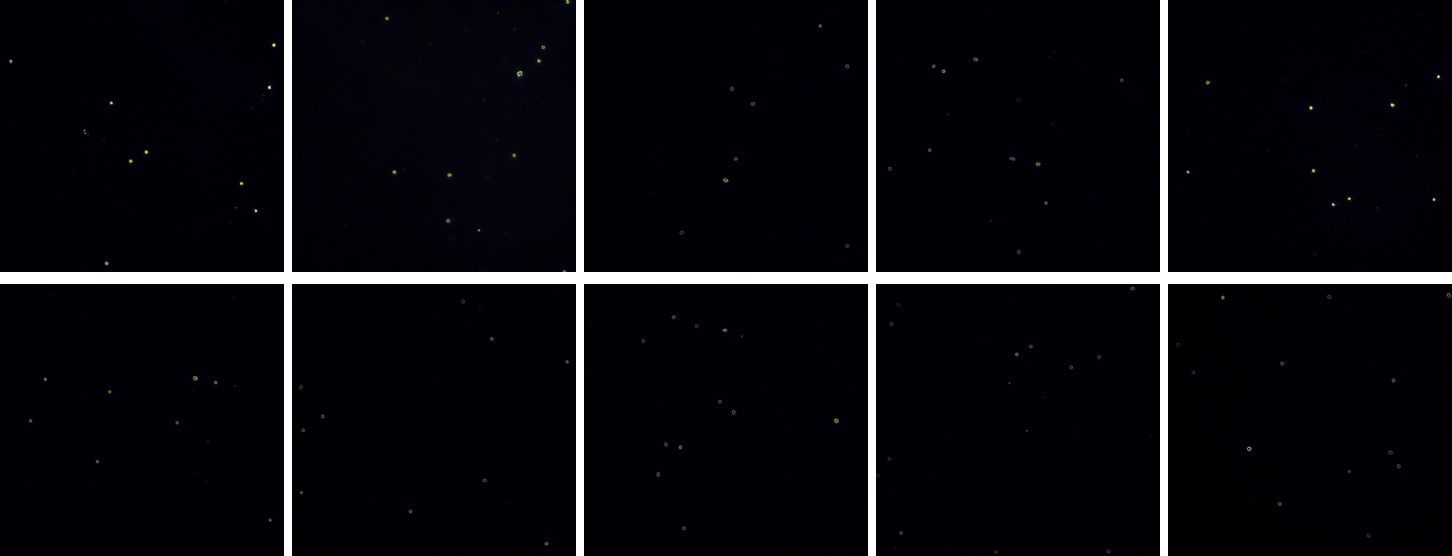
**

**Figure S7.**

**
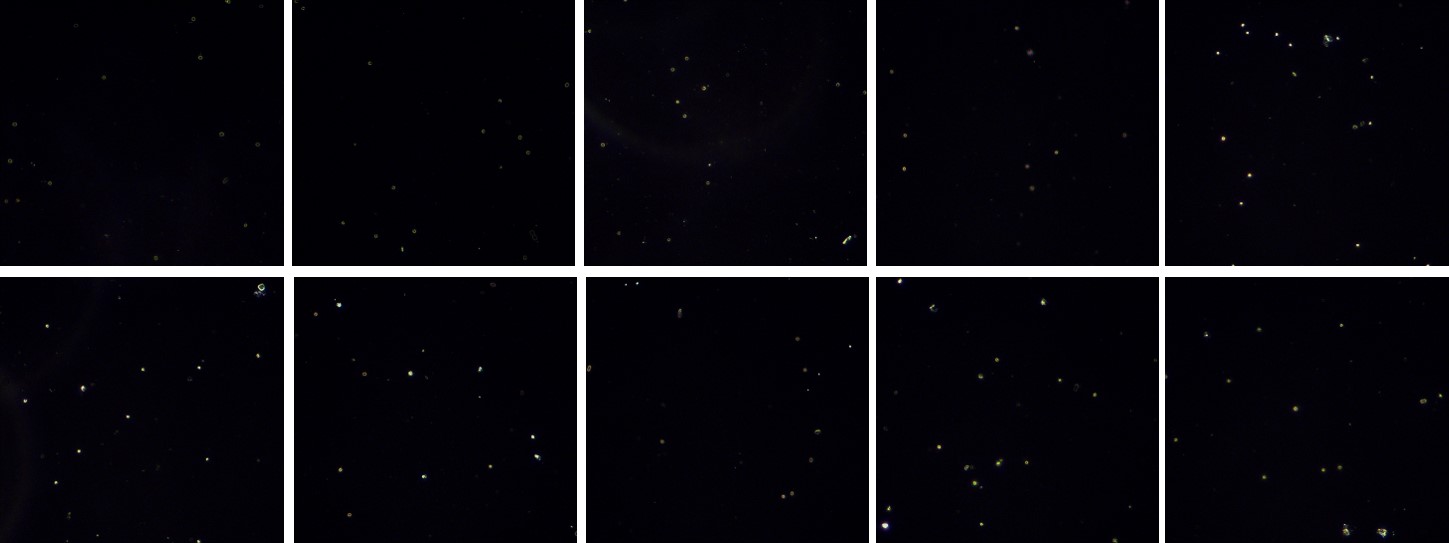
**

**Figure S8.**

**
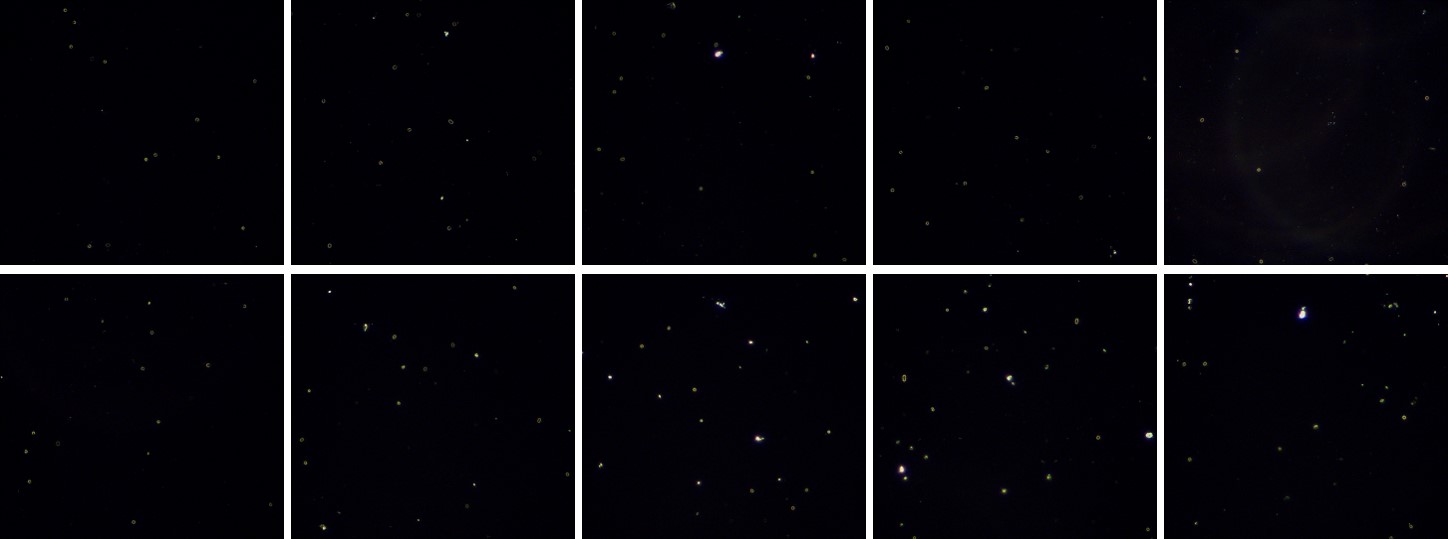
**

| **Samples** | **No. 1** | **No. 2** | **No. 3** | **No. 4** | **No. 5** | **No. 6** | **No. 7** | **No. 8** | **No. 9** | **No. 10** |
| --- | --- | --- | --- | --- | --- | --- | --- | --- | --- | --- |
| **Dilute soup** | 15 | 15 | 15 | 11 | 13 | 12 | 25 | 11 | 12 | 18 |
|  | 17 | 22 | 15 | 10 | 16 | 12 | 10 | 10 | 10 | 15 |
|  | 18 | 13 | 18 | 10 | 22 | 14 | 15 | 17 | 7 | 8 |
| **Rice** | 12 | 10 | 13 | 11 | 9 | 12 | 9 | 10 | 9 | 9 |
|  | 18 | 10 | 11 | 12 | 11 | 12 | 9 | 8 | 12 | 10 |
|  | 10 | 14 | 18 | 13 | 14 | 8 | 11 | 11 | 12 | 8 |

**Table S1. The number of counting result of one view (CFU)**
